# Enhancing Water Resistance in Foam Cement through MTES-Based Aerogel Impregnation

**DOI:** 10.3390/gels10020118

**Published:** 2024-02-01

**Authors:** Zhi Li, Shengjie Yao, Guichao Wang, Xi Deng, Fang Zhou, Xiaoxu Wu, Qiong Liu

**Affiliations:** School of Resources and Safety Engineering, Central South University, Changsha 410083, China

**Keywords:** silica aerogels, foam concrete, water resistance, softening coefficient, compressive strength, impregnation method

## Abstract

The propensity of foamed concrete to absorb water results in a consequential degradation of its performance attributes. Addressing this issue, the integration of aerogels presents a viable solution; however, their direct incorporation has been observed to compromise mechanical properties, attributable to the effects of the interface transition zone. This study explores the incorporation of MTES-based aerogels into foamed cement via an impregnation technique, examining variations in water–cement ratios. A comprehensive analysis was conducted, evaluating the influences of MTES-based aerogels on the thermal conductivity, compressive strength, density, chemical composition, and microstructure of the resultant composites across different water–cement ratios. Our findings elucidate that an increment in the water–cement ratio engenders a gradual regularization of the pore structure in foamed concrete, culminating in augmented porosity and diminished density. Notably, aerogel-enhanced foamed concrete (AEFC) exhibited a significant reduction in water absorption, quantified at 86% lower than its conventional foamed concrete (FC) counterpart. Furthermore, the softening coefficient of AEFC was observed to surpass 0.75, with peak values reaching approximately 0.9. These results substantiate that the impregnation of MTES-based aerogels into cementitious materials not only circumvents the decline in strength but also bolsters their hydrophobicity and water resistance, indirectly enhancing the serviceability and longevity of foamed concrete. In light of these findings, the impregnation method manifests promising potential for broadening the applications of aerogels in cement-based materials.

## 1. Introduction

Globally, the increasing energy demand has resulted in an energy crisis, the depletion of finite energy resources, and a myriad of environmental issues, such as ozone depletion, global warming, and climate change [1]. The demand for energy in buildings has surged in recent years, driven by population growth and higher living standards. Consequently, enhancing the energy efficiency of buildings can substantially decrease dependence on fossil fuels and play a pivotal role in mitigating overall greenhouse gas emissions [2]. At the current consumption rate, it is anticipated that non-renewable energy sources, such as oil and natural gas, will be exhausted in the first half of the 21st century. Research indicates that building energy consumption constitutes a significant portion, ranging from 30% to 50%, of the overall energy consumption in national economies [3,4]. Therefore, conserving energy in buildings represents a crucial measure to mitigate the energy crisis and promote the sustainable development of human society [5,6,7]. In the pursuit of energy conservation, incorporating thermal insulation materials into building envelopes offers a substantial opportunity. Compared to other renewable energy sources like solar and wind energy, building energy conservation delivers more tangible economic benefits. As a result, considerable attention has been directed towards developing thermal insulation materials as a strategy to achieve efficient energy savings and zero emissions in buildings. Research in this field has primarily focused on enhancing the thermal insulation properties of building envelopes to enhance their overall energy efficiency [8,9].

The use of aerogel in building insulation is gaining popularity due to its low thermal conductivity and excellent insulation performance. M. Koebel et al. [10] suggest that SAs have great promise for applications in the insulation market, but more research activities are still needed to improve aerogel insulation materials. Research has shown that a mere 20 mm of aerogel can reduce heat loss through building exterior walls by up to 90% [11,12]. Yan et al. conducted a study on the use of silica aerogel to improve the performance of rock wool and glass wool and the preparation of silica aerogel and composite insulation with expanded perlite for building insulation [13,14]. To further enhance the thermal insulation properties of concrete, S. Fickler incorporated silica aerogel particles into a high-strength cement matrix, resulting in high-performance aerogel concrete with a compressive strength ranging from 3.0 MPa to 23.6 MPa and a thermal conductivity ranging from 0.16 W/m/K to 0.37 W/m/K [15]. Similarly, Serina Ng added aerogel to ultra-high-performance concrete to create aerogel-containing mortar, which exhibited a compressive strength of 20 MPa and a thermal conductivity of 0.55 W/m/K with a 50% volume content of aerogel and a thermal conductivity of 0.31 W/m/K with an 80% volume content of aerogel. In comparison to ordinary mortar, a thinner insulation thickness was required for aerogel insulation mortar to achieve the same insulation effect [16]. Scholars have also investigated the influence of various factors on the thermal conductivity of aerogel mortar, and a comparative analysis suggested that aerogel-mortar thermal insulation systems were effective in reducing building energy consumption compared to vitrified-microsphere-mortar thermal insulation systems.

Silica aerogel (SA) is known for its hydrophobicity, which poses challenges when attempting to mix it with water-based cementitious materials. The formation of an interfacial transition zone between the aerogel and the water-based cementitious materials has a negative impact on the workability of the composite slurry [17]. To address this issue, many researchers have focused on how to eliminate the interface transition zone to improve interface bonding. For example, Cui et al. [18] found that modifying hydrophobic SiO_2_ aerogel with KH550 and epoxy resin AB glue can effectively eliminate the interface bonding area, leading to significant improvements in the compressive strength of the composite material. However, it is important to note that the composite material prepared using this method has poor thermal insulation performance and runs counter to the hydrophobic nature of the aerogel material [19].

Based on the above research, it can be inferred that the first problem to be solved is the composite of aerogel and geopolymer. The purpose of this study is to highlight the thermal insulation properties, mechanical properties, and water resistance of the composites obtained by incorporating a Methyltrichlorosilane (MTES)-based aerogel into foamed concrete using the impregnation method. The results obtained will contribute to the application of aerogel materials in the building sector.

## 2. Results and Discussion

### 2.1. Microstructure

**Figure 1:**
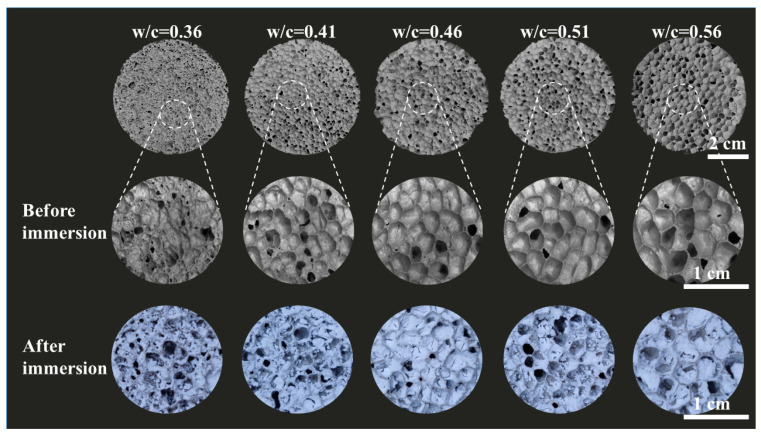
Pore structure changes before and after immersion of FC and AEFC samples with different water–cement ratios.

Figure 1 illustrates a clear trend wherein the pore size of the foamed concrete increases as the water–cement ratio (w/c) ascends from 0.36 to 0.56. Notably, at w/c ratios below 0.41, the FC exhibits a disorganized pore structure. However, as the w/c ratio exceeds 0.41, the pores progressively adopt a more circular shape, accompanied by a marginal increase in size. This observed phenomenon can be ascribed to the excess free water derived from the 3% hydrogen peroxide solution employed during the FC preparation. The ensuing chemical reactions during this process are delineated below.

(1) $$2nH_{2}O_{2}\frac{∆}{PH>7}2nH_{2}O+nO_{2}$$

The findings depicted in Figure 1 reveal that an increment in the water–cement ratio (w/c) corresponds to a gradual evolution in the pore morphology of the foamed concrete, transitioning from irregular to circular configurations. This morphological transformation becomes pronounced at a w/c ratio of 0.41. The viscosity of the cement slurry emerges as a critical determinant in shaping the pore structure, with high-viscosity slurries conducive to irregular pore formation and low-viscosity slurries favoring circular pores, as supported by the literature [20,21]. Moreover, the role of hydrogen peroxide in modulating pore size is noteworthy. An escalation in the w/c ratio marginally elevates the concentration of hydrogen peroxide, catalyzing the emergence of larger pores. Additionally, the incorporation of MTES-based aerogels into the FC matrix induces a pore-filling effect with aerogel materials, exerting a substantial impact on the physicochemical attributes of the resultant aerogel-embedded foamed concrete.

**Figure 2:**
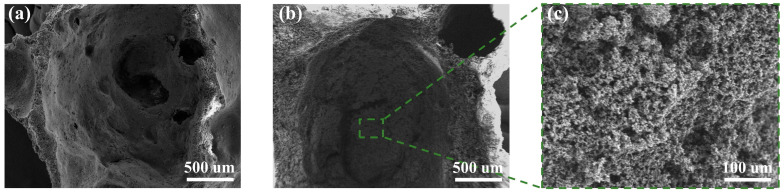
Pore structures of (**a**) FC and (**b**) AEFC; and (**c**) SEM images of MTES-based aerogels in AEFC.

Figure 2 presents scanning electron microscopy (SEM) images elucidating the pore structures of FC, AEFC, and the MTES-based aerogels within AEFC. Complementarily, Table 1 and Table 2 furnish energy dispersive spectroscopy (EDS) elemental surface analysis results corresponding to Figure 2a and Figure 2b, respectively. A notable distinction is observed in the textural comparison of FC and AEFC; the surface of AEFC depicted in Figure 2b is smoother relative to the rougher texture of FC in Figure 2a, a phenomenon attributable to the infill provided by MTES-based aerogels. Furthermore, Figure 2c distinctly showcases the typical coral-like silica framework structure of the MTES-based aerogel embedded within the pores of AEFC, as depicted in Figure 2b [22]. From Table 3, it can be seen that the cement Ca element is more heavily weighted, a comparative analysis of the EDS elemental surface data from Table 1 and Table 2 reveals a stark contrast in elemental composition. The data in Table 1 (corresponding to Figure 2a) indicate a predominant presence of calcium, reflective of the calcium salts integral to the cement matrix of FC. Conversely, Table 2 (pertaining to Figure 2b) exhibits a preponderance of silicon, predominantly ascribed to the MTES-based aerogels within AEFC. The contribution of hydrated calcium silicate to the silicon content within the cement matrix of AEFC is discerned to be relatively minor [23].

### 2.2. Chemical Composition and Hydrophobic Mechanism

**Figure 3:**
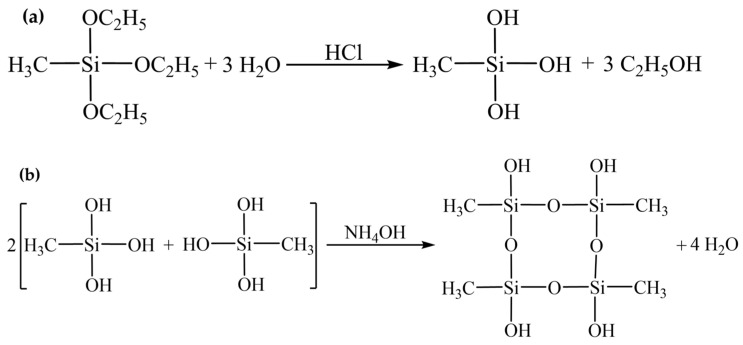
The synthetic procedure employed in this study involves hydrolysis (**a**) and condensation reactions (**b**).

The synthetic procedure employed in this study involves hydrolysis and condensation reactions, as delineated in Figure 3, respectively [24]. Notably, the Si-CH_3_ groups intrinsic to the MTES molecule and its hydrolysis byproducts remain unaltered throughout the hydrolysis process. These persistent Si-CH_3_ groups are ultimately integrated into the silica framework, where they significantly influence the surface chemistry of the MTES-based aerogels [25].

**Figure 4:**
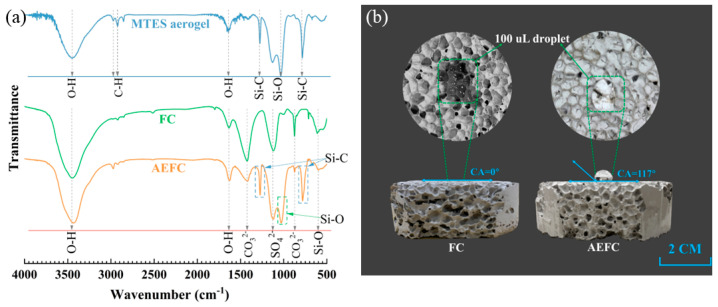
(**a**) FTIR spectra of MTES-based aerogel, FC, and AEFC; and (**b**) hydrophobicity of FC and AEFC.

Figure 4a showcases the Fourier-transform infrared (FTIR) spectroscopy analysis of MTES-based aerogel and FC. In the spectrum of the MTES-based aerogel, Si–O–Si bond asymmetric stretching vibration bands manifest in the ranges of 1010–1090 cm^−1^ and 465 cm^−1^ [23]. Peaks at 782 cm^−1^ and 1272 cm^−1^ signify stretching and symmetric deformation vibrations of the Si–C bonds [22,26]. Broad absorption at 3436 cm^−1^ and a peak around 1647 cm^−1^ correspond to –OH group vibrations [27], while peaks at 2902 cm^−1^ and 2964 cm^−1^ are indicative of C–H bond asymmetric and symmetric stretching vibrations [28]. These peaks highlight the Si–CH_3_ groups, which impart hydrophobicity to the aerogel.

In the FC spectrum, the peak at 3461 cm^−1^ is attributed to hydroxyl stretching vibrations of Ca(OH)_2_ [29], and the peak at 1646 cm^−1^ to deformation vibration in free water. Carbonate diffraction peaks at 1430 cm^−1^ and 876 cm^−1^ arise due to the reaction of CO_2_ and Ca(OH)_2_ forming CaCO_3_ [30]. The sulfate group’s antisymmetric stretching vibration is noted at 1120 cm^−1^ [31,32], and asymmetric tensile vibration peaks of Si–O at 1000 cm^−1^ and 521 cm^−1^ suggest the presence of calcium silicate hydrate [32].

The AEFC spectrum features all peaks present in both the MTES aerogel and FC, without any additional peaks, suggesting that no further chemical reactions occur between the MTES solution and FC during the impregnation process. This indicates that the MTES-based aerogel merely adheres to the surface of the FC substrate.

Figure 4b illustrates the hydrophobicity comparison between FC and AEFC by showcasing the behavior of 100 µL water droplets on their surfaces (fill light was applied during the photo shoot). The hydrophilic nature of FC is evident as the water droplet spreads across its surface. In contrast, AEFC exhibits markedly better hydrophobicity, with the water droplet maintaining a stable, spherical shape atop its surface (contact angle (CA) = 117°). This enhanced hydrophobicity can be attributed to the Si-CH_3_ groups present on the MTES-based aerogel, as elucidated in Figure 3a,b. These groups confer the aerogel with its inherent hydrophobic properties [22]. Post impregnation, the FC surface becomes coated with the MTES-based aerogel, effectively creating a hydrophobic layer. Consequently, AEFC exhibits a degree of hydrophobicity that is nearly on par with the MTES-based aerogel itself.

### 2.3. Water Resistance

**Figure 5:**
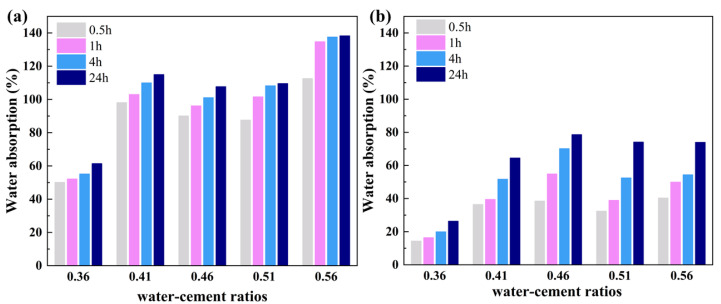
(**a**) Water absorption of FC at different times; (**b**) water absorption of AEFC at different times.

The durability of materials in aqueous or humid environments is crucial, necessitating the evaluation of post-immersion properties such as water absorption and softening coefficient [33,34,35]. Figure 5 delineates the influence of the water–cement ratio on the water absorption characteristics of FC and AEFC. Notably, the water absorption of both FC and AEFC escalates with the increment in the water–cement ratio until a plateau is reached beyond a ratio of 0.41. This phenomenon correlates with the morphological evolution of the pore structure from irregular to more uniformly circular pores at elevated water–cement ratios. However, a further increment in the water–cement ratio to 0.56 results in a substantial surge in the water absorption of FC, concomitant with a decline in material density and strength and an augmentation in porosity. Figure 5a substantiates that FC attains water absorption saturation after an hour of immersion. Conversely, AEFC shows significantly lower water absorption compared to FC, owing to the hydrophobic properties imparted by the MTES-based aerogel. Beyond a water–cement ratio of 0.41, AEFC’s water absorption stabilizes around 75%, marking an 86% reduction relative to FC. Additionally, AEFC exhibits a more protracted water absorption trajectory in comparison to FC.

**Figure 6:**
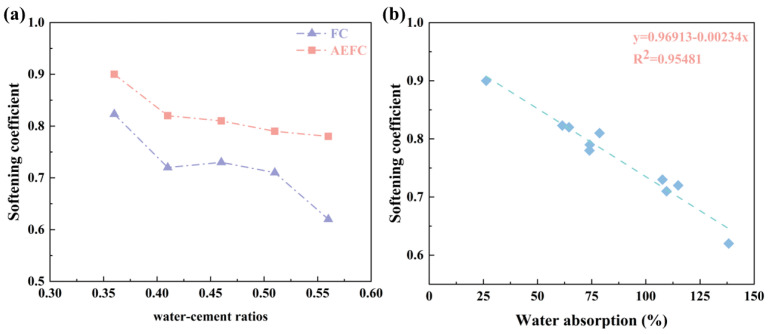
(**a**) Softening coefficient of FC and AEFC under different water–cement ratios; (**b**) relationship between water absorption and softening coefficient.

The softening coefficient is a pivotal metric for materials deployed in aqueous or humid conditions, serving as an indicator of their structural integrity post immersion. Standards dictate that materials subjected to prolonged damp conditions should exhibit a softening coefficient exceeding 0.85, while those in mildly humid environments should maintain a coefficient no less than 0.75 [33]. Figure 6 illustrates the variation in softening coefficients for FC and AEFC across different water–cement ratios, along with the correlation between water absorption and softening coefficient.

As depicted in Figure 6a, there is an evident decrement in the softening coefficients for both FC and AEFC concomitant with increasing water–cement ratios. Notably, FC exhibits a pronounced reduction in its softening coefficient upon reaching a water–cement ratio of 0.56. Conversely, AEFC consistently maintains higher softening coefficients compared to FC, with values surpassing the 0.75 threshold, reinforcing its suitability for use in moist environments. In response to the higher softening coefficient of FC with a w/c ratio of 0.46 compared to that with a w/c ratio of 0.41 observed in Figure 6a, we suggest this could be due to microstructural variances and experimental variability affecting mechanical properties. Further investigation is needed to fully understand these mechanisms.

Figure 6b reveals a linear relationship between the softening coefficient and water absorption ($y=0.96913-0.00234x;R^{2}=0.95481$), underscoring water absorption as a significant determinant of the softening coefficient. This linear correlation suggests that enhancing material hydrophobicity could be a strategic avenue to bolster the softening coefficient, thereby augmenting the durability of foamed concrete in wet conditions.

### 2.4. Compressive Strength

**Figure 7:**
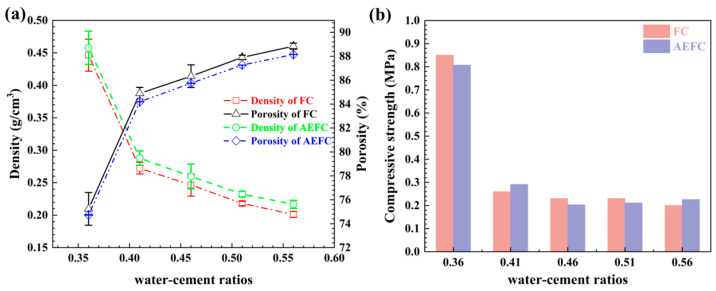
(**a**) Density and porosity of FC and AEFC; (**b**) compressive strength of FC and AEFC.

Figure 7a depicts the inverse relationship between water–cement ratio and density for both FC and AEFC before and after immersion treatment, in accordance with established microstructural dynamics [36,37]. Notably, at a water–cement ratio of 0.41, FC transitions to a regular circular pore structure. Post immersion, AEFC retains the same trend as FC but exhibits a higher density across corresponding water–cement ratios. This increased density in AEFC can be attributed to the incorporation of MTES-based aerogel, which slightly augments the mass without altering the volume. As per Equations (8) and (9), porosity and density are inversely proportional when matrix density is constant, leading to a slight reduction in porosity for AEFC compared to FC, as illustrated in Figure 7a.

Figure 7b reflects the congruent trends in compressive strength for AEFC and FC in relation to their densities. A gradual decline in compressive strength from 0.83 MPa to 0.28 MPa is observed with increasing water–cement ratio, followed by a stabilization in the 0.23–0.28 MPa range [38,39,40]. However, due to the conditions, we only made a single measurement of the sample. The incorporation of MTES-based aerogel in AEFC does not markedly influence its compressive strength. The decrease in strength is largely due to heightened porosity stemming from the shift in pore structure towards regular circular forms and the consequent enlargement of pores. It is a well-established fact that the strength of porous materials is inversely related to their porosity. However, AEFC benefits from a more uniform and orderly microstructure, which aids in the effective dispersion of stress, thereby stabilizing compressive strength within the specified range despite the increase in porosity.

### 2.5. Thermal Insulation Property

**Figure 8:**
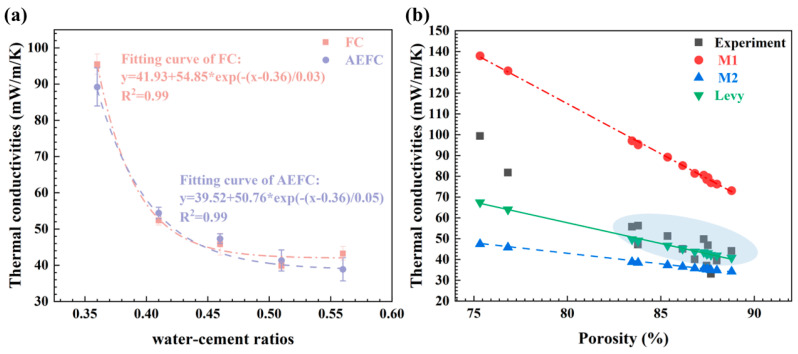
(**a**) Variation trend of thermal conductivity of FC and AEFC with water–cement ratios; and (**b**) comparison between experimental data and effective thermal conductivity calculated by different heat transfer models.

Figure 8a illustrates the thermal conductivity of FC and AEFC in relation to the water–cement ratio. Based on measurements of the effective thermal conductivity of the insulation material [41], for FC, the thermal conductivity diminishes significantly from 0.095 W/m/K to 0.038 W/m/K as the water–cement ratio increases from 0.36 to 0.56. This reduction is attributed to the sharp decrease in density and the corresponding increase in porosity from approximately 74% to 84–88%. In this porous structure, static air, with its low thermal conductivity of 0.026 W/m/K, predominates, thereby significantly lowering the effective thermal conductivity of the material.

Similar to FC, AEFC exhibits a corresponding decrease in thermal conductivity with increasing water–cement ratio, stabilizing at around 0.04 W/m/K [42,43,44]. Despite the incorporation of MTES-based aerogel, the thermal properties of AEFC are closely aligned with those of FC, mainly because the aerogel is only physically combined with FC, making their physical property differences negligible for the purposes of this analysis.

To model the effective thermal conductivity of composite materials like AEFC, three basic calculation models can be utilized, excluding the Wiener boundary, composed of series and parallel models [45]. The Maxwell–Eucken model, which has two variants, Maxwell–Eucken-1 (M1) and Maxwell–Eucken-2 (M2), is based on the distinction between the continuous phase and the dispersed phase [46,47,48,49,50,51].

AEFC is considered a ternary material in which the cementitious component is the only continuous phase and the MTES-based aerogel and gas are the dispersed phases. For the gas/MTES-based aerogel mixed phase, the gas can be considered the continuous phase and the MTES-based aerogel the dispersed phase, while the effective thermal conductivity of the gas/MTES-based aerogel mixed phase can be calculated by the Maxwell–Eucken-1 model [45,52].

(2) $$K_{a,g}=K_{g}\frac{2K_{g}+K_{a}-2(K_{g}-K_{a})v_{a}}{2K_{g}+K_{a}+(K_{g}-K_{a})v_{a}}$$

$K_{g}$ and $K_{a}$ represent the thermal conductivities of gas and the MTES-based aerogel, respectively. The thermal conductivity of stationary air is typically 26 mW/m/K. The volume fraction of SA in the gas/SA mixed phase is represented by $v_{a}$.

When the cement base is in the continuous phase, the overall thermal conductivity $k_{c,a,g}$ is represented by the M1 model as follows:

(3) $$k_{c,a,g}=K_{c}\frac{2K_{c}+K_{a,g}-2(K_{c}-{K_{a}}_{,g}){v_{a}}_{,g}}{2K_{c}+{K_{a}}_{,g}+(K_{c}-{K_{a}}_{,g}){v_{a}}_{,g}}$$

When the gas/MTES-based aerogel mixture is in the continuous phase, it is expressed by M2 as follows:

(4) $$k_{c,a,g}=K_{a,g}\frac{2K_{a,g}+K_{c}-2(K_{a,g}-K_{c})v_{c}}{2K_{a,g}+K_{c}+(K_{a,g}-K_{c})v_{c}}$$

However, the limitation of the M1 and M2 models is that they are determined only based on the difference between the dispersed phase and the continuous phase and lack relevant correction parameters. The relatively accurate representation of the Levy model is shown as follows [52]:

(5) $$K=K_{C}\frac{2K_{C}+K_{a,g}-2(K_{C}-K_{a,g})F}{2K_{C}+K_{a,g}+(K_{C}-K_{a,g})F}$$

where K, $K_{c}$, $K_{a,g}$ are the effective thermal conductivity of composite materials, the cement thermal conductivity, and the gas/SA mixed-phase thermal conductivity, respectively. The revised parameter expressions are as follows:

(6) $$F=\frac{2/G-1+2v_{a,g}-\sqrt{(2/G-1+2v_{a,g}{)}^{2}-8v_{a,g}/G}}{2}$$

where $v_{a,g}$ is the pore volume fraction, namely porosity, and parameter G is expressed as follows:

(7) $$G=\frac{(K_{C}-K_{a,g}{)}^{2}}{(K_{C}+K_{a,g}{)}^{2}+K_{C}K_{a,g}/2}$$

The comparison of the effective thermal conductivity values calculated using models M1 and M2 and the Levy model with the experimental data for foam concrete and aerated foam concrete is depicted in Figure 8b. At lower porosities, the experimental thermal conductivities for FC and AEFC lie between the values predicted by M1 and the Levy model. For higher porosities, the experimental thermal conductivities of FC and AEFC are in close agreement with those predicted by the Levy model, as indicated by the blue ellipse in Figure 8b. As shown in Figure 1, the sample exhibits an irregular pore structure and low porosity at a water–cement ratio of 0.36. When this ratio exceeds 0.41, the porosity of the sample surges rapidly to over 84%, and the pores in FC begin transitioning into circular forms. This transition is a critical factor influencing the alignment of experimental thermal conductivity values with those predicted by the Levy model. Consequently, it can be inferred that the circular pore structure in FC, as illustrated in Figure 1, correlates more closely with the Levy model’s calculations. However, when the pore structure alters, further modifications are required.

## 3. Conclusions

In this work, the feasibility of fabricating aerogel-enhanced foamed concrete using varying water-to-cement ratios through the impregnation technique was explored. The results elucidate that the water-to-cement ratio is pivotal in dictating the microstructural attributes of the foamed concrete, specifically its pore configuration and porosity. With a fixed foam stabilizer content, reduced water-to-cement ratios were associated with a more chaotic pore structure due to the resultant increase in slurry viscosity. In contrast, a ratio of 0.41 yielded moderate viscosity, which allowed the foam stabilizer to sustain the bubble structure more effectively, resulting in a more organized pore framework. Furthermore, a significant dependency of the physical characteristics of AEFC on the water-to-cement ratio was observed. An increase in this ratio led to an escalation in porosity and a concomitant reduction in density to as low as 0.2 g/cm^3^, while maintaining a direct and positive correlation with compressive strength. Importantly, our study demonstrated the material’s enhanced hydrophobic properties with a remarkable 86% decrease in water absorption at a water-to-cement ratio of 0.56 compared to regular foamed concrete, surpassing the minimum standard for the softening coefficient by registering an increase from 0.62 to 0.78. These findings highlight the potential of water-to-cement ratio manipulation as a strategic tool to enhance the waterproofing characteristics of aerogel-enhanced foamed concrete, indicating its suitability for applications requiring specific structural and waterproofing criteria.

## 4. Materials and Methods

### 4.1. Raw Materials

Methyl trichlorosilane (MTES, 98%) and cetyltrimethylammonium bromide (CTAB, 99%) purchased from Shanghai Aladdin Biochemical Technology Co., Ltd. (Shanghai, China) were used as the precursor and surfactant, respectively. Hydrochloric acid (HCl, 36–38%) and ammonium hydroxide (NH_3_·H_2_O, 25–28%) were purchased from Sinopharm Chemical Reagent Co., Ltd. (SCRC, Shanghai, China) and used as acid and base catalysts, respectively. The sulfur aluminate cement (R. SAC42.5) used in this experiment was obtained from Zibo Yunhe Color Cement Co., Ltd. (Zibo, Shandong Province, China). The chemical composition of cement is shown in Table 3. Calcium stearate, which acts as a stabilizer in foamed cement, was provided by Hebei Baiyilian Chemical Co., Ltd. (Shijiazhuang, Hebei Province, China). Hydrogen peroxide solution (H_2_O_2_, 3%), which is a foaming agent, was sourced from Jiangxi Grass Coral Disinfection Products Co., Ltd. (Nanchang, Jiangxi Province, China).

### 4.2. Sample Preparation

#### 4.2.1. Preparing Foamed Cement

Initially, calcium stearate and sulfur aluminate cement were dry-mixed in a mass ratio of 1:293. Subsequently, 3 wt.% hydrogen peroxide solution was added to the powder and heated to 40 ± 2 °C. The water–cement ratio was controlled by adjusting the amount of hydrogen peroxide solution in the range of 0.36–0.56. The heated mixture was then stirred rapidly and poured into a mold, followed by placement in a 60 °C water bath to accelerate the decomposition of hydrogen peroxide and achieve the initial setting. The resulting foamed concrete was cured in an environment with 95% RH and a temperature of 20 ± 2 °C. After curing, the foamed concrete was dried at 60 °C for 1 day and then further dried up to a constant weight at 80 °C. This method was utilized to investigate the preparation of foamed concrete with controlled water–cement ratios, aiming to establish a new method for developing high-performance cement-based composites.

#### 4.2.2. Preparing MTES-Based Aerogel

Initially, a mixture of 5 mL MTES, 0.01 g CTAB, 25 mL DI·H_2_O, and 300 μL 0.1 M HCl was stirred for 3 min in a 100 mL beaker. The resulting hybrid solution was kept in a 45 °C water bath and stirred for approximately 2 h to undergo hydrolysis. After hydrolysis, the hybrid solution was cooled to below 10 °C to regulate the condensation rate, based on the Arrhenius equation. Subsequently, 500 μL of 1 M NH_3_·H_2_O was added to the hybrid solution while stirring for 10 s. The resulting gel was placed in an incubator below 10 °C; gelation typically occurs within 1.5 h [22].

#### 4.2.3. Embedding of MTES-Based Aerogel

**Figure 9:**
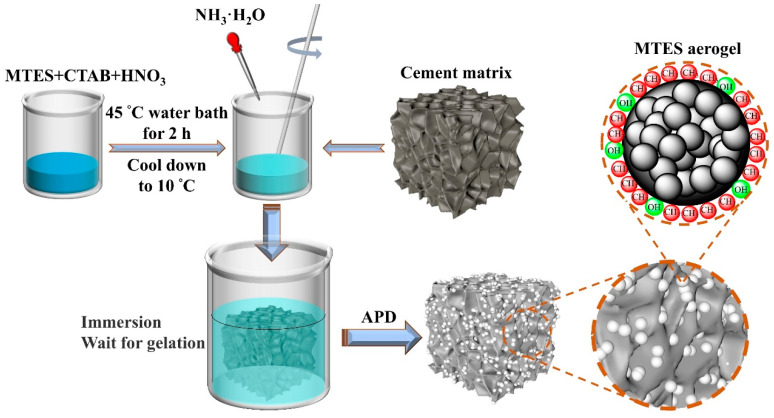
Schematic diagram of the experimental flow.

During the preparation of the MTES-based aerogel, the above-mentioned foamed concrete was completely immersed in the MTES hydrolyzed solution after adding the 1 M NH_3_·H_2_O. Subsequently, the formed sol with foamed cement was placed in an incubator below 10 °C; the gelation usually occurs within 1.5 h. (It is important to note that although FC may absorb a small amount of moisture, this moisture, as well as moisture in the gel, is removed during the drying process). Finally, the composites were dried under ambient pressure at 120 °C for 3 h to obtain the MTES-based aerogel/foamed concrete composite (aerogel-embedded foamed concrete, AEFC). A schematic diagram of the experimental flow is shown in Figure 9.

### 4.3. Methods of Characterization

The internal microstructure of the material was examined using a German ZEISS Sigma 300 field emission scope (SEM) (Oberkochen, Germany) and its accompanying equipment, including energy dispersive spectroscopy (EDS).

The bulk density ($\rho_{b}$) was approximated through tap density, which was measured using a tap density meter (ZS-202, Liaoning Instrument Research Institute Co., Ltd. Liaoning, China) with 300 r/min for continuous vibration within 10 min. The corresponding porosity (the gas volume fraction, $v_{g}$) was calculated from the following formula [53]:

(8) $$porosity=\left(1-\frac{\rho_{b}}{\rho_{m}}\right)\times 100\%$$

In this study, cement and SA are considered together as the solid-phase matrix of AFC, and the matrix density ($\rho_{m}$) is calculated as follows:

(9) $$\rho_{m}=1/(w_{c}/\rho_{c}+w_{a}/\rho_{a})$$

where $\rho_{c}$, $\rho_{a}$ and $w_{c}$, $w_{a}$ correspond to the density and mass fraction of cement and the MTES-based aerogel–silicon framework, respectively; the density is 1.803 g/cm^3^ and 2.25 g/cm^3^, respectively [54].

The chemical bonds and chemical groups of pure SA, FC, and AFC were determined by Fourier-transform infrared spectroscopy (FTIR, Nicolet 8700, Nicolet, Madison, WI, USA) using KBr pellets.

The hydrophobicity of the samples was measured using the ASR-705S hydrophobic angle tester from Guangdong Iris Instrument Technology Co., Ltd. (Dongguan, China), with contact angles recorded and obtained via the image processing program ImageJ (version: 1.54f) [55].

To measure water absorption, we first conditioned standard and aerogel-enhanced foamed concrete samples to a stable weight, then weighed them dry. After full immersion in distilled water for different periods of time, we removed surface moisture and re-weighed the samples. The water absorption rate was calculated as (Wet Weight − Dry Weight)/Dry Weight × 100%, providing insights into changes in water absorption due to aerogel incorporation.

The compressive strength testing was conducted using a column sensor produced by Ningbo Saishi Measurement and Control Technology Co., Ltd. (Ningbo, China) under a loading rate of 0.5 mm/min. The single compressive test conducted for each sample adhered to “JG/T 266-2011”.

The thermal conductivity of the samples was determined at room temperature and ambient pressure using a thermal conductivity meter (XIATECH, TC3000E probe, China).

## Figures and Tables

**Table 1:** EDS surface analysis of element-specific gravity of FC.

Element	Weight%	Atomic %	Weight Error %	Net Int.	K Ratio
Si	6.76	9.37	7.66	59.17	0.0649
Ca	93.24	90.63	3.87	245.70	0.9245

**Table 2:** EDS surface analysis of element-specific gravity of AEFC.

Element	Weight %	Atomic %	Weight Error %	Net Int.	K Ratio
Si	96.16	97.27	2.74	639.21	0.9599
Ca	3.84	2.73	25.75	6.65	0.0343

**Table 3:** Chemical composition of cement.

Oxide Constituents	SO_3_	CaO	Al_2_O_3_	SiO_2_	Fe_2_O_3_	MgO	Na_2_O	K_2_O	TiO_2_	Else
Content (wt.%)	16.95	59.90	13.04	4.00	2.43	0.93	0.24	0.45	0.85	1.21

## Data Availability

The data presented in this study are openly available in article.

## References

[1] Pérez-Lombard L., Ortiz J., Pout C. (2008). A Review on Buildings Energy Consumption Information. Energy Build..

[2] Pasupathy A., Velraj R., Seeniraj R.V. (2008). Phase Change Material-Based Building Architecture for Thermal Management in Residential and Commercial Establishments. Renew. Sustain. Energy Rev..

[3] Berardi U. (2018). Aerogel-Enhanced Systems for Building Energy Retrofits: Insights from a Case Study. Energy Build..

[4] Pacheco Torgal F., Buratti C., Kalaiselvam S., Granqvist C.-G., Ivanov V. (2016). Nano and Biotech Based Materials for Energy Building Efficiency.

[5] Zhang Z., Li B., Wang Z., Liu W., Liu X. (2023). Development of Reduced Thermal Conductivity Ductile Cement-Based Composite Material by Using Silica Aerogel and Silane. J. Build. Eng..

[6] Mehta A., Ashish D.K. (2020). Silica Fume and Waste Glass in Cement Concrete Production: A Review. J. Build. Eng..

[7] Rudžionis Ž., Adhikary S.K., Manhanga F.C., Ashish D.K., Ivanauskas R., Stelmokaitis G., Navickas A.A. (2021). Natural Zeolite Powder in Cementitious Composites and Its Application as Heavy Metal Absorbents. J. Build. Eng..

[8] Adhikary S.K., Ashish D.K. (2022). Turning Waste Expanded Polystyrene into Lightweight Aggregate: Towards Sustainable Construction Industry. Sci. Total Environ..

[9] Berardi U. (2017). A Cross-Country Comparison of the Building Energy Consumptions and Their Trends. Resour. Conserv. Recycl..

[10] Koebel M., Rigacci A., Achard P. (2012). Aerogel-Based Thermal Superinsulation: An Overview. J. Sol-Gel Sci. Technol..

[11] Cuce E., Cuce P.M., Wood C.J., Riffat S.B. (2014). Optimizing Insulation Thickness and Analysing Environmental Impacts of Aerogel-Based Thermal Superinsulation in Buildings. Energy Build..

[12] Cuce E., Cuce P.M., Wood C.J., Riffat S.B. (2014). Toward Aerogel Based Thermal Superinsulation in Buildings: A Comprehensive Review. Renew. Sustain. Energy Rev..

[13] Yan Q., Meng Z., Luo J., Wu Z. (2021). Experimental Study on Improving the Properties of Rock Wool and Glass Wool by Silica Aerogel. Energy Build..

[14] Yan Q., Feng Z., Luo J., Xia W. (2022). Preparation and Characterization of Building Insulation Material Based on SiO_2_ Aerogel and Its Composite with Expanded Perlite. Energy Build..

[15] Fickler S., Milow B., Ratke L., Schnellenbach-Held M., Welsch T. (2015). Development of High Performance Aerogel Concrete. Energy Procedia.

[16] Ng S., Sandberg L.I.C., Jelle B.P. (2014). Insulating and Strength Properties of an Aerogel-Incorporated Mortar Based an UHPC Formulations. Key Eng. Mater..

[17] Sobolev K., Flores I., Torres-Martinez L.M., Valdez P.L., Zarazua E., Cuellar E.L., Bittnar Z., Bartos P.J.M., Němeček J., Šmilauer V., Zeman J. (2009). Engineering of SiO_2_ Nanoparticles for Optimal Performance in Nano Cement-Based Materials. Nanotechnology in Construction 3.

[18] Cui Y., Wang D., Zhao J., Li D., Liu Z., Ng S. (2019). Thermal and Mechanical Properties of SiO_2_ Aerogel–Incorporated Geopolymer Insulation Materials. J. Mater. Civ. Eng..

[19] Adhikary S.K., Ashish D.K., Rudžionis Ž. (2021). Aerogel Based Thermal Insulating Cementitious Composites: A Review. Energy Build..

[20] Jiang J., Lu Z., Niu Y., Li J., Zhang Y. (2016). Study on the Preparation and Properties of High-Porosity Foamed Concretes Based on Ordinary Portland Cement. Mater. Des..

[21] Meng T., Wei H., Yang X., Zhang B., Zhang Y., Zhang C. (2021). Effect of Mixed Recycled Aggregate on the Mechanical Strength and Microstructure of Concrete under Different Water Cement Ratios. Materials.

[22] Deng X., Wu L., Deng Y., Huang S., Sun M., Wang X., Liu Q., Li M., Li Z. (2021). Effects of Precursor Concentration on the Physicochemical Properties of Ambient-Pressure-Dried MTES Based Aerogels with Using Pure Water as the Only Solvent. J. Sol-Gel Sci. Technol..

[23] Al-Oweini R., El-Rassy H. (2009). Synthesis and Characterization by FTIR Spectroscopy of Silica Aerogels Prepared Using Several Si(OR)_4_ and R″Si(OR′)_3_ Precursors. J. Mol. Struct..

[24] Nadargi D.Y., Latthe S.S., Hirashima H., Rao A.V. (2009). Studies on Rheological Properties of Methyltriethoxysilane (MTES) Based Flexible Superhydrophobic Silica Aerogels. Microporous Mesoporous Mater..

[25] Li Z., Zhao S., Koebel M.M., Malfait W.J. (2020). Silica Aerogels with Tailored Chemical Functionality. Mater. Des..

[26] Socrates G. (2004). Infrared and Raman Characteristic Group Frequencies: Tables and Charts.

[27] Li Z., Cheng X., He S., Shi X., Yang H. (2015). Characteristics of Ambient-Pressure-Dried Aerogels Synthesized via Different Surface Modification Methods. J. Sol-Gel Sci. Technol..

[28] Huang S., Wu X., Li Z., Shi L., Zhang Y., Liu Q. (2020). Rapid Synthesis and Characterization of Monolithic Ambient Pressure Dried MTMS Aerogels in Pure Water. J. Porous Mater..

[29] El-Alfi E.A., Gado R.A. (2016). Preparation of Calcium Sulfoaluminate-Belite Cement from Marble Sludge Waste. Constr. Build. Mater..

[30] García-Maté M., De la Torre A.G., León-Reina L., Losilla E.R., Aranda M.A.G., Santacruz I. (2015). Effect of Calcium Sulfate Source on the Hydration of Calcium Sulfoaluminate Eco-Cement. Cem. Concr. Compos..

[31] Ylmén R., Jäglid U., Steenari B.-M., Panas I. (2009). Early Hydration and Setting of Portland Cement Monitored by IR, SEM and Vicat Techniques. Cem. Concr. Res..

[32] Scrivener K.L. (2004). Backscattered Electron Imaging of Cementitious Microstructures: Understanding and Quantification. Cem. Concr. Compos..

[33] Li T., Huang F., Li L., Zhu J., Jiang X., Huang Y. (2020). Preparation and Properties of Sulphoaluminate Cement-Based Foamed Concrete with High Performance. Constr. Build. Mater..

[34] Yoon H.-S., Lim T.-K., Jeong S.-M., Yang K.-H. (2020). Thermal Transfer and Moisture Resistances of Nano-Aerogel-Embedded Foam Concrete. Constr. Build. Mater..

[35] Iwański M.M., Chomicz-Kowalska A., Maciejewski K. (2020). Resistance to Moisture-Induced Damage of Half-Warm-Mix Asphalt Concrete with Foamed Bitumen. Materials.

[36] Sharma R., Kim H., Lee N.K., Park J.-J., Jang J.G. (2023). Microstructural Characteristics and CO_2_ Uptake of Calcium Sulfoaluminate Cement by Carbonation Curing at Different Water-to-Cement Ratios. Cem. Concr. Res..

[37] Haruehansapong S., Pulngern T., Chucheepsakul S. (2014). Effect of the Particle Size of Nanosilica on the Compressive Strength and the Optimum Replacement Content of Cement Mortar Containing Nano-SiO_2_. Constr. Build. Mater..

[38] Gavela S., Nikoloutsopoulos N., Papadakos G., Passa D., Sotiropoulou A. (2018). Multifactorial Experimental Analysis of Concrete Compressive Strength as a Function of Time and Water-to-Cement Ratio. Procedia Struct. Integr..

[39] Wang F., Li K., Liu Y. (2022). Optimal Water-Cement Ratio of Cement-Stabilized Soil. Constr. Build. Mater..

[40] Rahmani K., Rahmanzadeh B., Piroti S. (2018). Experimental Study of the Effect of Water-Cement Ratio on Compressive Strength, Abrasion Resistance, Porosity and Permeability of Nano Silica Concrete. Frat. Integrità Strutt..

[41] Yan Q., Shen X., Luo J., Yan H., Gao H. (2019). Experimental Study on Effective Thermal Conductivity of Building Insulation Materials. Meas. Sci. Technol..

[42] Sang G., Zhu Y., Yang G., Zhang H. (2015). Preparation and Characterization of High Porosity Cement-Based Foam Material. Constr. Build. Mater..

[43] Liu Z., Zhao K., Hu C., Tang Y. (2016). Effect of Water-Cement Ratio on Pore Structure and Strength of Foam Concrete. Adv. Mater. Sci. Eng..

[44] Wang L., Liu P., Jing Q., Liu Y., Wang W., Zhang Y., Li Z. (2018). Strength Properties and Thermal Conductivity of Concrete with the Addition of Expanded Perlite Filled with Aerogel. Constr. Build. Mater..

[45] Lee J.-H., Lee S.-H., Choi C., Jang S., Choi S. (2010). A Review of Thermal Conductivity Data, Mechanisms and Models for Nanofluids. Int. J. Micro-Nano Scale Transp..

[46] Kaddouri W., El Moumen A., Kanit T., Madani S., Imad A. (2016). On the Effect of Inclusion Shape on Effective Thermal Conductivity of Heterogeneous Materials. Mech. Mater..

[47] Chauhan D., Singhvi N., Singh R. (2013). Dependence of Effective Thermal Conductivity of Composite Materials on the Size of Filler Particles. J. Reinf. Plast. Compos..

[48] Khan K.A., Khan S.Z., Khan M.A. (2016). Effective Thermal Conductivity of Two-Phase Composites Containing Highly Conductive Inclusions. J. Reinf. Plast. Compos..

[49] Pal R. (2007). New Models for Thermal Conductivity of Particulate Composites. J. Reinf. Plast. Compos..

[50] Wang J., Carson J.K., North M.F., Cleland D.J. (2006). A New Approach to Modelling the Effective Thermal Conductivity of Heterogeneous Materials. Int. J. Heat Mass Transf..

[51] Chu Z. (2023). Combination of the Unifying Model for the Effective Thermal Conductivity of Isotropic, Porous and Composite Geomaterials. Int. J. Rock Mech. Min. Sci..

[52] Levy F.L. (1981). A Modified Maxwell-Eucken Equation for Calculating the Thermal Conductivity of Two-Component Solutions or Mixtures. Int. J. Refrig..

[53] Luo Y., Li Z., Zhang W., Yan H., Wang Y., Li M., Liu Q. (2019). Rapid Synthesis and Characterization of Ambient Pressure Dried Monolithic Silica Aerogels in Ethanol/Water Co-Solvent System. J. Non-Cryst. Solids.

[54] Li Z., Cheng X., He S., Shi X., Yang H., Zhang H. (2016). Tailoring Thermal Properties of Ambient Pressure Dried MTMS/TEOS Co-Precursor Aerogels. Mater. Lett..

[55] Kistler S.S. (2002). Coherent Expanded-Aerogels. J. Phys. Chem..

